# Special collection in association with the 2023 International Conference on aging, innovation and rehabilitation

**DOI:** 10.1186/s12938-024-01243-x

**Published:** 2024-05-21

**Authors:** Babak Taati, Milos R. Popovic

**Affiliations:** Toronto, Canada

We are happy to introduce this collection of research articles in association with the International Conference on aging, innovation and rehabilitation (ICAIR). The 2023 conference was an interdisciplinary event that brought together leading researchers, scientists, and entrepreneurs, dedicated to enhancing the quality of life for individuals who face challenges related to aging and disability (Fig. 1). The conference was jointly hosted by The KITE Research Institute | Toronto Rehabilitation Institute–University Health Network (Fig. 2) and the Rehabilitation Sciences Institute at the University of Toronto, with contributions and participation from other clinical and research institutions and hospitals worldwide.

**Fig. 1:**
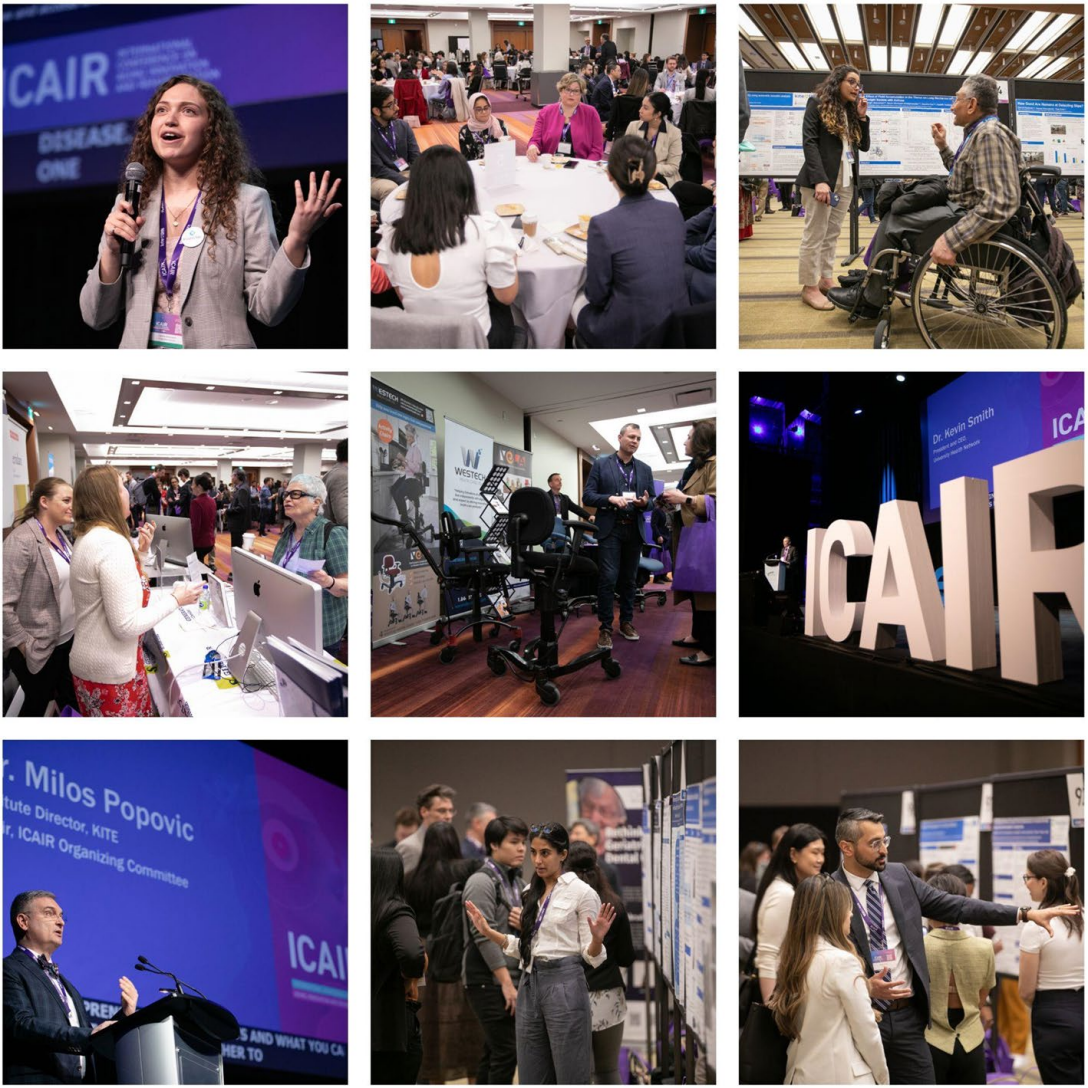
Snapshots from the first International Conference on Aging, Innovation and Rehabilitation (ICAIR), May 2023, Toronto, Ontario, Canada

**Fig. 2:**
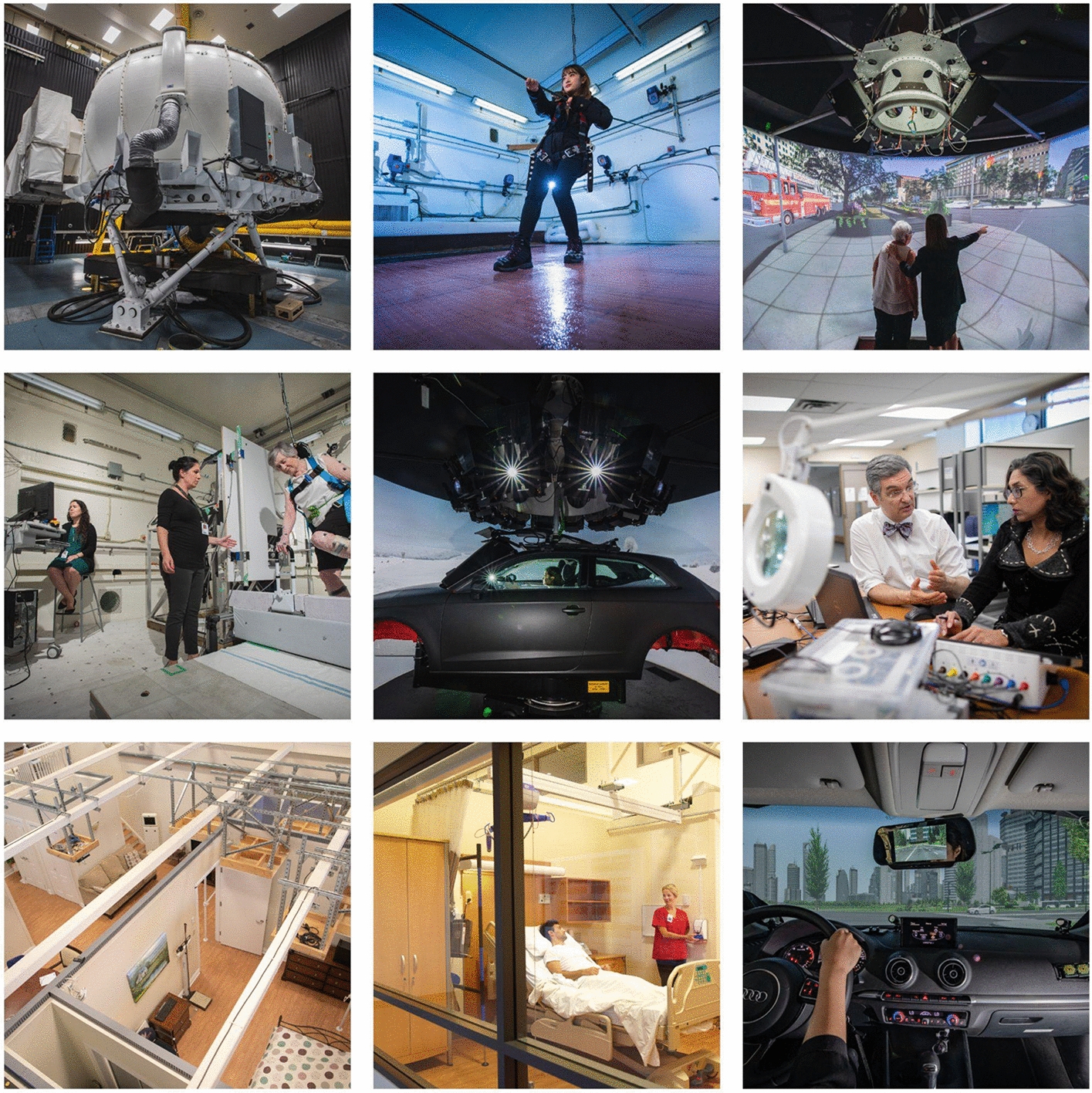
Toronto Rehabilitation Institute is Canada’s largest academic hospital dedicated to adult rehabilitation and complex continuing care and a member of the University Health Network, a network of research hospitals affiliated with the University of Toronto. KITE Research Institute is the research arm of the Toronto Rehabilitation Institute–University Health Network. KITE is a world leader in complex rehabilitation science and is dedicated to improving the lives of people living with the effects of disability, illness and aging. KITE’s areas of focus include prevention, restoration, enhanced participation, and independent living. *KITE research facilities include the DriverLab, ClimateLab, CareLab, Rehab Engineering Lab, StreetLab, *etc

Abstracts submitted to the conference underwent peer review process and were selected for poster or podium presentations. A small subset of the abstracts, which received the highest review scores, were invited to submit a full-length manuscript for review and potential publication in this collection. These submissions underwent standard peer-review process at the journal and, after reviews, rebuttals, and revisions, twelve were accepted for publication, representing a wide range of techniques and applications related to health monitoring, assessment, and rehabilitation.

While covering diverse topics, articles in this collection are linked through multiple connecting themes, such as functional electrical stimulation [1, 2] or the application of signal processing and artificial intelligence in solving aging and rehabilitation problems [2–8]. Specifically, a number of the papers in this collection [4–7] explore the application of computer vision techniques in various healthcare domains, particularly focusing on rehabilitation and mobility assistance. Lim et al. [2], for instance, investigate the feasibility of using depth cameras and pressure mats in a balance training system for individuals with spinal cord injuries. A previous pilot study had shown the potential of a visual-feedback balance training (VFBT), coupled with closed-loop functional electrical stimulation (FES), to improve the standing balance in individuals with incomplete spinal-cord injury/disease [9, 10]. However, clinical implementation of such systems would be limited because of the required force plates, which are expensive and not easily accessible. Lim et al. [2] experimentally demonstrate that depth cameras and pressure mats can accurately track the body center of mass and center of pressure. As another example, Sabo et al. [7] demonstrate the responsiveness of a previously developed predictive vision-based machine learning model [11, 12] to measure changes in gait in response to medication and deep brain stimulation in individuals with Parkinson’s disease.

Following the success of ICAIR 2023, the conference will run as an annual event, each May in Toronto, Ontario, Canada. We plan to continue this collection in future, and each year invite a selection of some of the most exciting research projects presented at ICAIR.

## Data Availability

Not applicable.
